# ‘Setting the Benchmark’ Part 4: Contextualising the Match Demands of Teams at the FIFA Women’s World Cup Australia and New Zealand 2023

**DOI:** 10.5114/biolsport.2025.142638

**Published:** 2024-08-30

**Authors:** Paul S. Bradley

**Affiliations:** 1 FIFA, Zürich, Switzerland

**Keywords:** Soccer, Female, Evolution, Physical, International, Team, Work Rate, Matches

## Abstract

The aims of the present study were to: (1) analyse the upper and lower match physical performance benchmarks and variability of teams at the FIFA Women’s World Cup Australia and New Zealand 2023, (2) examine the evolving team sprint ranking across three Women’s World Cups and (3) investigate noteworthy relationships between collective physical and tactical metrics. With FIFA’s official approval, all sixty-four games at the tournament were analysed using an optical tracking system alongside FIFA’s Enhanced Football Intelligence metrics. On average, teams at the FIFA Women’s World Cup 2023 covered 103.3 ± 4.4 km in total, with 6.7 ± 0.6 km and 1.9 ± 0.3 km covered at the higher intensities (≥19.0 & ≥23.0 km · h^-1^), respectively. The top five ranked teams from a high-intensity running perspective (Zambia, Spain, Brazil, Canada, Denmark) covered 24–44% more distance than the bottom five ranked teams (Jamaica, Columbia, Costa Rica, Switzerland, Vietnam) at the tournament (*P* < 0.01; Effect Size [ES]: 2.3–2.5). Match-to-match variation of teams revealed Italy and Panama were particularly consistent for the distances covered at higher intensities (Coefficient of Variation [CV]: 0.3–4.5%), while Costa Rica demonstrated considerable variation (CV: 23.4–40.7%). Teams generally covered more total distance on a per-minute basis in the first versus the second half (*P* < 0.01; ES: 1.1), but no differences existed at higher intensities (*P* > 0.05; ES: 0.1–0.2). Correlations were found between the number of high-intensity runs and various phase of play events for defensive transitions and recoveries, in addition to progressions up the pitch and into the final third (*r* = 0.48–0.88; *P* < 0.01). A basic comparative analysis revealed Spain demonstrated the most pronounced increase (2015 = 9^th^, 2019 = 35^th^, 2023 = 90^th^ percentile; CV: 92.6%) and China PR the most marked decrease (2015 = 22^nd^, 2019 = 30^th^, 2023 = 0 percentile; CV: 89.6%) in their sprinting percentile rank across the last three FIFA Women’s World Cups. The present findings provide a depiction of the current collective demands of international women’s football. This information could be useful for practitioners to benchmark team performances and to potentially understand the myriad of contextual factors impacting physical performances.

## INTRODUCTION

The popularity of women’s football has increased in recent years, with both the number of registered players and spectators continuing to grow worldwide [1]. Despite such progress, the women’s game is still at an embryonic stage in relation to scientific research coverage [2]. Thus, an essential starting point for research exploration in this area could be to comprehensively evaluate the match demands of women’s football. This would not only inform future research questions, but could also provide a framework for the development of female-specific training [3, 4].

Although quantifying the demands of match-play are complex, researchers commonly report the distance covered in total and at higher intensities [5]. Despite the limitations of such an approach (e.g., provides ‘what’ distance was covered but not ‘why’ it was covered from a tactical perspective), this information can still serve as an important point of reference for practitioners regarding physical benchmarks [6, 7]. For instance, establishing upper and lower physical benchmarks of contemporary football allows appropriate comparisons to be made across both the short- and long-term. Investigations that establish such normative profiles typically describe the activity patterns of elite female players at a positional level [8–10]. However, limited information exists on the match demands at a team level in contemporary international tournaments. Thus, in accordance with the primary aim of the present study, teams at the FIFA Women’s World Cup Australia and New Zealand 2023 were benchmarked to provide an accurate depiction of the current collective demands of international women’s football. Since a high degree of variation in movement profiles exists in elite female players across various playing positions [11], it would also be of interest to quantify match-to-match variation on a team level at such an international tournament (i.e., average, range and outliers).

Research demonstrates that elite female players cover less distance in the second half compared to the first half of matches across a range of physical metrics [3]. Thus, the intense, intermittent nature of women’s football may cause some degree of physiological fatigue towards the end of the game [9, 10]. However, football is a complex sport and second-half performance declines are not only related to fatigue [12]. For instance, match importance and the changing game state are among a multitude of factors that can influence a team’s physical output [13]. Another aspect to consider is the introduction of new directives that have resulted in longer games that could further contribute to the accumulative fatigue experienced by teams across single and multiple games [7]. The latest rules also allowed teams to make five substitutions at the FIFA Women’s World Cup 2023, compared to just three at the 2015 and 2019 editions in Canada and France respectively. Alternatively, more substitutions could potentially offset the decline in the physical performance in the second half of longer matches at a team level. Although this is not a stated aim of the present study, it would still be of interest to examine half-by-half trends across teams to establish whether workrate distribution has changed due to modified rules.

The evolution of the match demands in men’s football has been extensively reported over the last decade in both domestic and international competitions [7, 14, 15]. This evidence indicates that the total distance covered during matches remains relatively static, while the distance covered at higher intensities continues to increase over time. Regarding women’s football, preliminary investigations revealed no differences in the match demands at the FIFA Women’s World Cup 2011 and 2015 editions [16]. However, the intensity of match-play increased by around 20–30% at the FIFA Women’s World Cup 2019 compared to previous tournaments [17]. At the FIFA Women’s World Cup 2023, some major changes occurred to both the rules and the technology used to quantify the match demands. This makes it challenging for researchers to establish if this performance ascent has continued in women’s international football. Due to such complexities, an alternative approach could be to track the physical rankings of each nation across multiple tournaments. Thus, the secondary aim of the present study was to track the team sprint ranking across three Women’s World Cups to provide practitioners with longitudinal insights into which countries have physically evolved more relative to other nations.

A myriad of factors can up or downregulate the match physical performances of teams and this can include the standard of opposition, match location, competition phase and the previous results leading up to the game [13]. Additional factors that potentially modulate match physical performances are often difficult to assess as the game is multifaceted in nature, whereby the technical, tactical, physical and psychological factors amalgamate during a match [12]. However, one of the most powerful modulating factors influencing a team’s physical output is the tactical approach that they employ [7, 18]. Recent research has found noteworthy associations between physical-tactical-technical metrics in men’s competitions [19], although such relationships have yet to be determined in the women’s game. Thus, the third aim of the present study was to use a correlational approach between various physical variables and FIFA’s Enhanced Football Intelligence metrics to provide much-needed context as to why teams physically exerted themselves during FIFA Women’s World Cup 2023 matches. Such contextual information may be valuable to practitioners and enable them to fully grasp the sheer complexity surrounding the match demands of women’s football. Moreover, gaining a deeper understanding of the inter-play between performance factors could enhance the replication of match scenarios within a training context.

Therefore, the aims of the present study were to: (1) analyse the upper and lower match physical performance benchmarks and variability of teams at the FIFA Women’s World Cup Australia and New Zealand 2023, (2) examine the evolving team sprint ranking across three Women’s World Cups and (3) investigate noteworthy relationships between physical and tactical metrics at a team level.

## MATERIALS AND METHODS

### Study Design and Scope

In this observational study, team trends were analysed at the FIFA Women’s World Cup Australia and New Zealand 2023 in an attempt to answer the following questions: What are the physical benchmarks of contemporary international female teams and how do these differ at the upper and lower extremes? Can collective match-to-match variability trends be established across various physical metrics (e.g., mean, range and outliers)? How do the sprint rankings of nations change across multiple tournaments? Is it possible to use FIFA’s Enhanced Football Intelligence metrics to identify the modulatory factors that up or downregulate the match physical demands of teams?

### Sample

This study analysed the match demands of all teams participating in the FIFA Women’s World Cup 2023. Team analyses involved the summation of match data of outfield players, including substitutes (goalkeeper data excluded). Matches were filtered so only full game data plus added time were evaluated (extra time data omitted). This format was reproduced in accordance with the official FIFA physical analyses of previous World Cups [7, 16, 17]. The sample consisted of 32 nations across 64 game observations. To the authors knowledge, all data are freely available [20], and thus no ethical approval was required. If any data presented are not available, then this could be shared by the author upon a reasonable and well justified request.

### Match Analysis System

With FIFA’s official approval, all Women’s World Cup 2023 games were analysed using an optical tracking system that operated at 25 Hz (TRACAB, Chyron Hego, Sweden). This systems validity has been quantified to verify the capture process and subsequent accuracy of the data [21]. After system calibration and various quality control processes, the data captured were analysed using match analysis software. This produced a data set on each team’s activity pattern during a match using female-specific speed zones [17].

### Speed Zones

Teams’ activities were coded into the following:

- Zone 1 (0.0–6.9 km · h^-1^)
- Zone 2 (≥7.0–12.9 km · h^-1^)
- Zone 3 (≥13.0–18.9 km · h^-1^)
- Zone 4 (≥19.0–22.9 km · h^-1^)
- Zone 5 (≥23.0 km · h^-1^)

Team analyses primarily reported physical metrics that provided insights into the volume (total distance) and intensity of match-play (high-intensity and sprinting distance or frequency counts). Total distance represented the sum of the ground covered in all speed zones. High-intensity distance and frequency counts consisted of the aggregation of zones 4 and 5 (≥19.0 km · h^-1^), while sprinting exclusively included zone 5 activity (≥23.0 km · h^-1^). Additionally, these metrics were analysed based on possession status or if the ball was out of play.

### FIFA’s Enhanced Football Intelligence Metrics

FIFA’s Enhanced Football Intelligence metrics were quantified [22], specifically the phases of play events that captured the tactical behaviours of teams during games. All phases of play were automatically identified through the optical tracking data using various computerised algorithms. FIFA’s algorithms broke these down into (1) in-possession phases and (2) out-of-possession phases. Seven inpossession phases were obtained in the initial analysis (build up, progression, final third, attacking transition, counter-attack, long ball and set piece) and the number of times teams performed them during matches were derived from the optical tracking data. Similarly, the occurrence of nine out-of-possession phases (low, mid, high block and press, counter-press, recovery and defensive transition) were initially obtained for further analysis using the optical tracking data obtained from the previously described system. For both in- and out-of-possession phases, the computerised algorithms used various features (spatial and physical) to identify and classify each phase automatically. For instance, it extracted ball and player pitch locations in relation to each other, in addition to the speed and direction of play. If teams entered a certain phase of play for a selected period of time, then the algorithms recorded this as a frequency count or an accumulated fraction of in-possession or out-of-possession time. The phases of play definitions can be found in freely available documentation [22].

### Evolving Team Sprint Ranking Across Three FIFA Women’s World Cups

The speed zones at the FIFA Women’s World Cup 2023 were identical to those at the Canada 2015 and France 2019 editions [17], but the latter two utilised a different optical tracking system (STATS LLC, USA). Thus, it was not possible to compare the physical performances of teams between tournaments. Consequently, teams that had participated in these three FIFA Women’s World Cups had their within tournament sprinting performances ranked into percentiles. This enabled evolving team sprinting performances to be identified relative to other nations within each tournament. As the evolution of match physical performances are more pronounced at zone 5 [14, 17], only sprinting distance as a percentage of total distance was evaluated in this way (e.g., sprint distance was divided into the total distance and then multiplied by 100).

### Statistical Analyses

Analyses were conducted using statistical software (IBM SPSS Statistics for Windows, Version 26.0. Armonk, NY). Descriptive statistics were calculated on each variable. To verify normality, z-scores were obtained through dividing the skewness and kurtosis values by their standard error. Differences between top and bottom ranked teams for various physical metrics were determined using independent *t*-tests. Team half-by-half differences for numerous physical metrics were assessed using paired-samples *t*-tests. Statistical significance was set at *P* < 0.05. Quadrant plot analysis composed of a simple percentage distribution computation [7], while basic percentile ranks were calculated using a standard function array formula. The coefficient of variation (CV) was calculated to determine the data spread across each metric (mean, range and outliers). Cohen’s effect sizes (ES) were computed to determine the meaningfulness of any differences. The ES magnitudes were classed as trivial (<0.2), small (>0.2–0.6), moderate (>0.6–1.2), and large (>1.2). Pearson’s correlation coefficients were used to determine any noteworthy relationships between physical and tactical metrics. The magnitudes of the associations were regarded as trivial (*r* ≤ 0.1), small (*r* > 0.1–0.3), moderate (*r* > 0.3–0.5), large (*r* > 0.5–0.7), very large (*r* > 0.7–0.9), and nearly perfect (*r* > 0.9). Values are presented as means and standard deviations unless otherwise stated.

## RESULTS

*Benchmarking & Variation*

On average, teams at the FIFA Women’s World Cup Australia and New Zealand 2023 covered 103.3 ± 4.4 km in total, with 6.7 ± 0.6 km and 1.9 ± 0.3 km covered at the higher intensities (≥19.0 & ≥23.0 km · h^-1^), respectively. The physical performances of teams at the upper and lower extremes also revealed some interesting trends. For instance, Figure 1A demonstrates that the top five ranked teams for total distance (Japan, Philippines, Demark, Norway, Zambia) covered 10–17% more ground in games compared to the bottom five ranked teams (Nigeria, Jamaica, Vietnam, Columbia, Portugal) at the tournament (*P* < 0.01; ES: 1.5–5.2). In Figure 1B, it is noteworthy that the top five ranked teams from a high-intensity running perspective (Zambia, Spain, Brazil, Canada, Denmark) covered 24–44% more distance than the bottom five ranked teams (Jamaica, Columbia, Costa Rica, Switzerland, Vietnam) at the competition (*P* < 0.01; ES: 2.3–2.5). Figure 1C illustrates that the top five ranked sprinting teams (Zambia, Spain, Brazil, United States, Australia) covered 41–78% more distance than the bottom five ranked teams (Switzerland, Vietnam, Republic of Ireland, Costa Rica, China PR) at the tournament (*P* < 0.01; ES: 3.0–4.4). To adhere to the primary aim of the study and for conciseness, only the physical performance variables are reported at the upper and lower extremes (e.g., top vs bottom trends) and thus technical and tactical trends were omitted.

**FIG. 1A:**
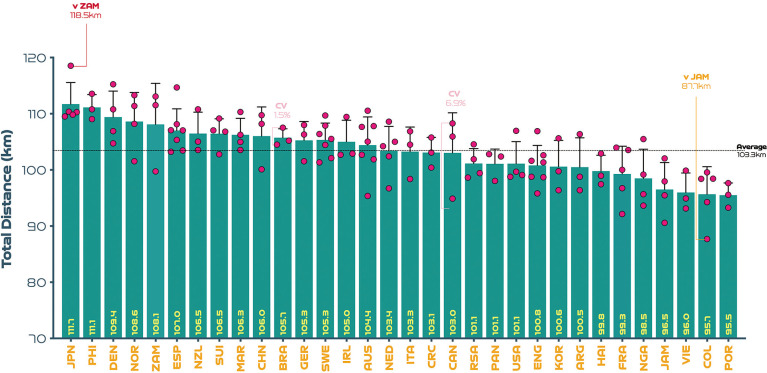
Total team distance: average per game and variation at the FIFA Women’s World Cup 2023. Data normalized for 90+ min (excludes goalkeepers and extra time). Red = max. Orange = min. Pink = high and low variation. Only outlier variation values are included (see text for CV average and range values).

**FIG. 1B:**
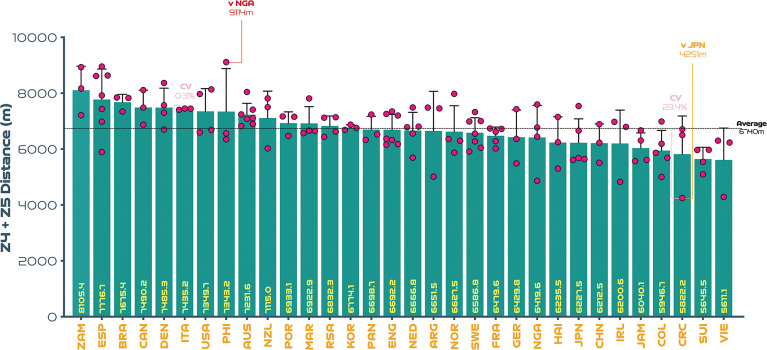
High-intensity team distance (Zones 4 + 5 [Z4 + Z5], ≥19.0 km · h^-1^): average per game and variation at the FIFA Women’s World Cup 2023. Data normalized for 90+ min (excludes goalkeepers and extra time). Red = max. Orange = min. Pink = high and low variation. Only outlier variation values are included (see text for CV average and range values).

Figures 1A–C also depict the performances of each nation per game as dots and their spread provides a basic visual representation of the match-to-match variation of each team at the FIFA Women’s World Cup 2023 (includes all group and knockout games). Although for conciseness only the average, range and outliers were reported as CV’s in the present analysis. On average, match-to-match CV’s during the tournament for total distance and that covered at the higher intensities (≥19.0 & ≥23.0 km · h^-1^) were 3.9% (range: 1.5–6.9%), 11.1% (range: 0.3–23.4%) and 18.8% (range: 4.5–40.7%), respectively. The most consistent team from a physical perspective was highly dependent on the metric. For instance, Brazil, Italy and Panama were particularly consistent for total distance (CV: 1.5%), high-intensity distance (CV: 0.3%) and sprint distance (CV: 4.5%), respectively. It is noteworthy that Canada exhibited the most variation from match-to-match for the distance covered in total (CV: 6.9%) and Costa Rica for that covered at high-intensity (CV: 23.4%) and sprinting (CV: 40.7%).

**FIG. 1C:**
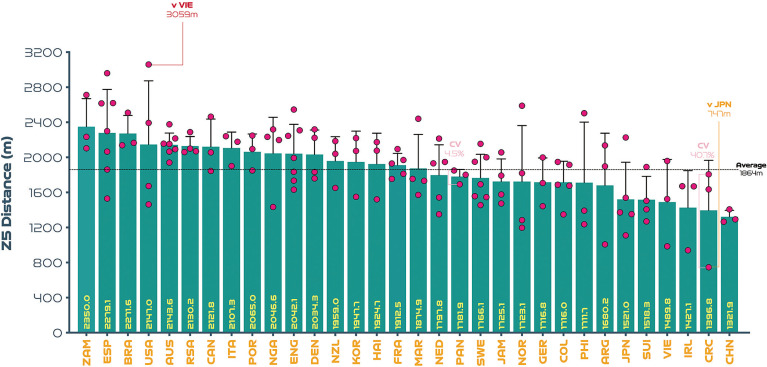
Sprint team distance (Zone 5 [Z5], ≥23.0 km · h^-1^): average per game and variation at the FIFA Women’s World Cup 2023. Data normalized for 90+ min (excludes goalkeepers and extra time). Red = max. Orange = min. Pink = high and low variation. Only outlier variation values are included (see text for CV average and range values).

## Quadrant Plots

Figure 2A correlates two distinct dimensions of team physical performance to determine the degree of association between the total and high-intensity distance covered. Cross-hairs denote the tournament average and are included to create quadrants that indicate which teams are more volume- or intensity-based. A moderate positive association was found between a team’s total and high-intensity game distances (*r* = 0.39; *P* < 0.05). Approximately 31% of the teams were in the lower-left quadrant, indicating that their volume and intensity characteristics were both low (Argentina, Colombia, Costa Rica, England, France, Haiti, Jamaica, Nigeria, Panama and Vietnam). Around 25% of teams were in the lower-right quadrant, indicating that they displayed a high volume but low intensity (China PR, Germany, Japan, the Netherlands, Norway, the Republic of Ireland, Sweden and Switzerland). Meanwhile, 19% of the teams were in the upper-left quadrant, which was indicative of a low volume but high intensity (Canada, Italy, Korea Republic, Portugal, South Africa and the USA). Finally, 25% of teams were in the upper-right quadrant, meaning that both their volume and their intensity were high (Australia, Brazil, Denmark, Morocco, New Zealand, the Philippines, Spain and Zambia).

**FIG. 2A:**
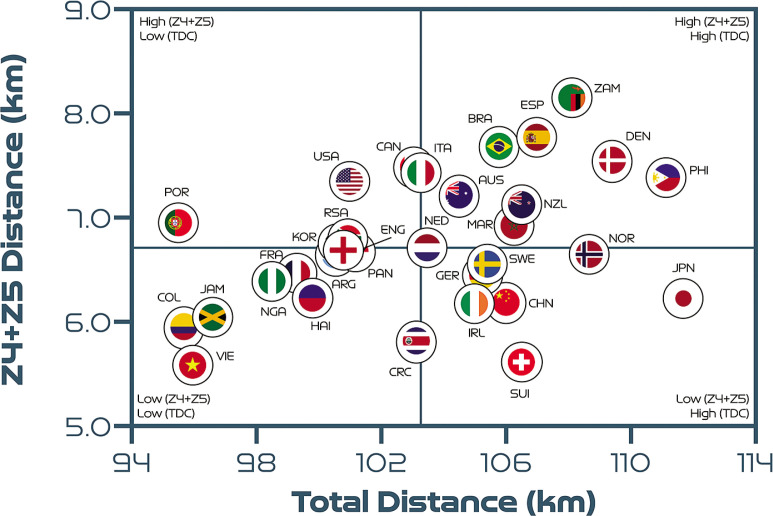
Total distance covered (TDC) versus high-intensity distance (Zones 4 + 5 [Z4 + Z5], ≥19.0 km · h^-1^) per team at the FIFA Women’s World Cup 2023. Data normalised for 90+ minutes (excludes goalkeepers and extra time). Crosshairs are based on the average for all teams. Correlation: *r* = 0.39; *P* < 0.05.

Figure 2B demonstrates no association between a team’s total and sprint distances (*r* = -0.03; *P* > 0.05). It was found that 19% of teams were in the lower-left quadrant, 28% were in the lowerright quadrant, 31% were in the upper-left quadrant and 22% were in the upper-right quadrant. The trends in Figures 2A and 2B are similar, as most teams can be found in the same quadrants. However, England, France, Haiti and Nigeria moved up from the lowerleft quadrant in Figure 2A to the upper-left quadrant in Figure 2B due to their elevated sprint distances. Moreover, the Philippines moved down from the upper-right quadrant in Figure 2A to the lower-right quadrant in Figure 2B due to their low sprint distance.

**FIG. 2B:**
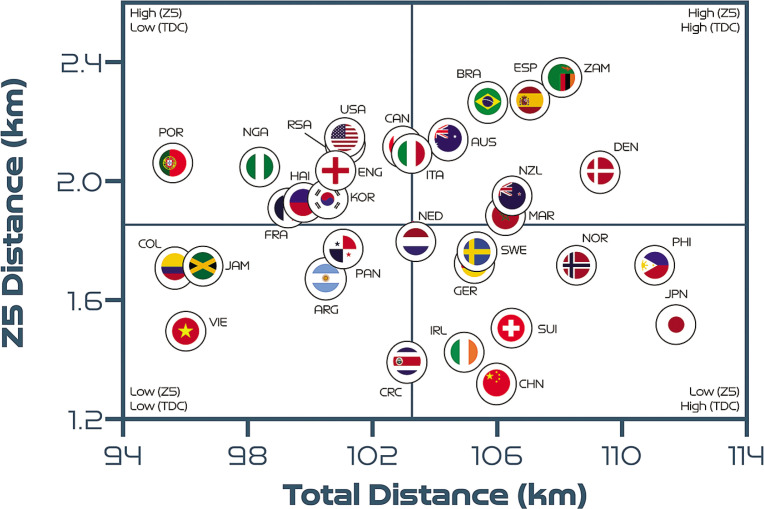
Total distance covered (TDC) versus sprint distance (Zone 5 [Z5], ≥23.0 km · h^-1^) per team at the FIFA Women’s World Cup 2023. Data normalised for 90+ minutes (excludes goalkeepers and extra time). Crosshairs are based on the average for all teams. Correlation: *r* = -0.03; *P* > 0.05.

## Half-by-Half Comparisons

Figure 3A highlights that teams generally covered less total distance on a per-minute basis in the second half than in the first half (*P* < 0.01; ES: 1.1). Germany and China PR demonstrated the most pronounced declines in total distance in the second half of matches on a per-minute basis. By contrast, Costa Rica, Vietnam, Jamaica and Argentina were the only nations that covered similar or higher total distances in the second half compared to the first period. However, Figures 3B and 3C demonstrate that no half-by-half deficits existed for the distance covered on a per-minute basis at higher intensities at the FIFA Women’s World Cup 2023 (*P* > 0.05; ES: 0.1–0.2). Brazil and Canada are striking examples of teams that displayed more intensity in the second half compared to the first half. In contrast, Vietnam and South Africa demonstrated markedly lower intensity in the second half compared to the first half.

**FIG. 3A:**
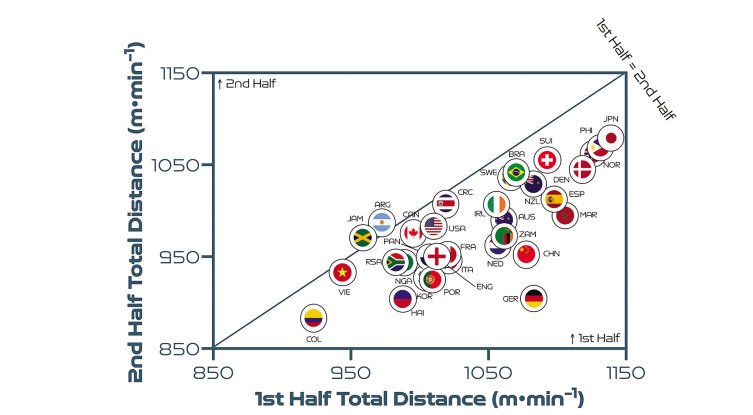
Team half-by-half total distance per minute at the FIFA Women’s World Cup 2023. Data normalized per minute and for 90+ min (excludes goalkeepers and extra time). Teams on the centre line covered the same distances in the 1^st^& 2^nd^ half. Teams in the bottom triangle covered more distance in the 1^st^ half and teams in top triangle covered more in the 2^nd^ half.

**FIG. 3B:**
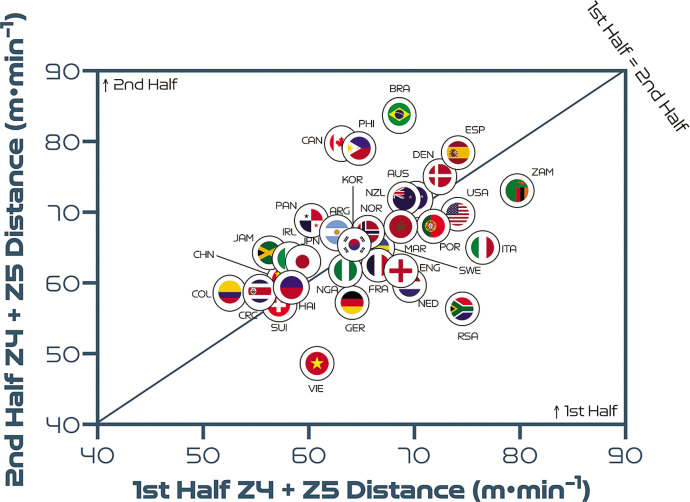
Team half-by-half high-intensity distance (Zones 4 + 5 [Z4 + Z5], ≥19.0 km · h^-1^) per minute at the FIFA Women’s World Cup 2023. Data normalized per minute and for 90+ min (excludes goalkeepers and extra time). Teams on the centre line covered the same distances in the 1^st^ & 2^nd^ half. Teams in the bottom triangle covered more distance in the 1^st^ half and teams in top triangle covered more in the 2^nd^ half.

**FIG. 3C:**
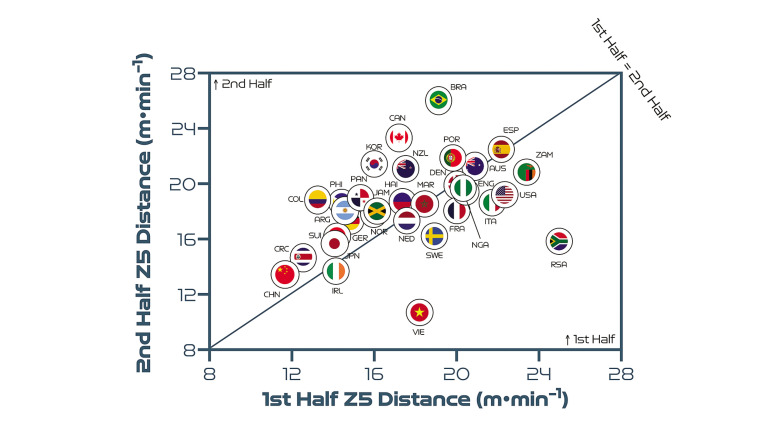
Team half-by-half sprint distance (Zone 5 [Z5], ≥23.0 km · h^-1^) per minute at the FIFA Women’s World Cup 2023. Data normalized per minute and for 90+ min (excludes goalkeepers and extra time). Teams on the centre line covered the same distances in the 1^st^ & 2^nd^ half. Teams in the bottom triangle covered more distance in the 1^st^ half and teams in top triangle covered more in the 2^nd^ half.

## FIFA’s Enhanced Football Intelligence Metrics

Regarding phases of play, Figure 4A indicates a moderate correlation between the number of high-intensity runs out-of-possession versus the combined number of events for defensive recoveries and transitions (*r* = 0.48; *P* < 0.01). Debutantes the Philippines, Zambia and Panama clearly resided within the upper-right quadrant, performing a plentiful number of each type of action.

**FIG. 4A:**
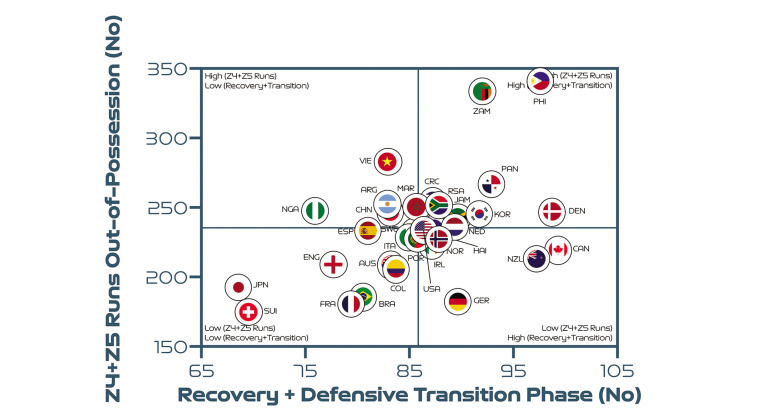
Team recovery and defensive transition phase count versus out-of-possession high-intensity runs (Zones 4 + 5 [Z4 + Z5], ≥19.0 km · h^-1^) at the FIFA Women’s World Cup 2023. Data normalized for 90+ min (excludes goalkeepers and extra time). Correlation; *r* = 0.48; *P* < 0.01.

Similarly, a very large association was found between the number of high-intensity runs performed in-possession and the number of progression and final-third phase of play events (*r* = 0.86–0.88; *P* < 0.01). In Figure 4B more attacking teams were found in the upper-right quadrant, such as Brazil, Spain and the USA, as they progressed up the pitch and into the final third more frequently than deeper, more defensive teams in the lower-left quadrant, such as Costa Rica, the Philippines and Vietnam.

**FIG. 4B:**
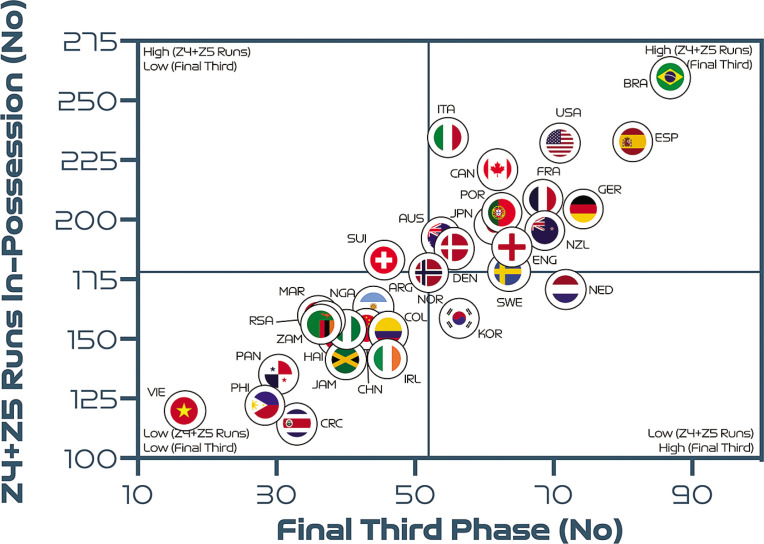
Team final third phase count versus high-intensity runs (Zones 4 + 5 [Z4 + Z5], ≥19.0 km · h^-1^). Data normalized for 90+min (excludes goalkeepers and extra time). Correlation; *r* = 0.86; *P* < 0.01.

## Evolving Team Sprint Ranking Across Three FIFA Women’s World Cups

Figure 5 displays the sprinting distances of teams across the last three Women’s World Cups as a percentage of total distance (Figure 5A) and also as a percentile rank (Figure 5B). Due to optical tracking changes across tournaments more emphasis should be placed on Figure 5B as this highlights teams that increased, decreased or maintained their sprinting percentile rank across multiple competitions. Noteworthy, examples of teams that substantially increased their sprinting percentile rank across three FIFA Women’s World Cups included Spain (2015 = 9^th^, 2019 = 35^th^, 2023 = 90^th^ percentile; CV: 92.6%) and Korea Republic (2015 = 4^th^, 2019 = 4^th^, 2023 = 65^th^ percentile; CV: 144.7%). In contrast, China PR (2015 = 22^nd^, 2019 = 30^th^, 2023 = 0 percentile; CV: 89.6%) and New Zealand (2015 = 74^th^, 2019 = 22^nd^, 2023 = 52^nd^ percentile; CV: 52.9%) decreased their sprinting percentile rank markedly across the three FIFA Women’s World Cups. Teams demonstrating a consistent percentile rank for sprinting across tournaments included France (2015 = 65^th^, 2019 = 52^nd^, 2023 = 58^th^ percentile; CV: 11.0%) and Canada (2015 = 87^th^, 2019 = 70^th^, 2023 = 77^th^ percentile; CV: 11.2%).

**FIG. 5A:**
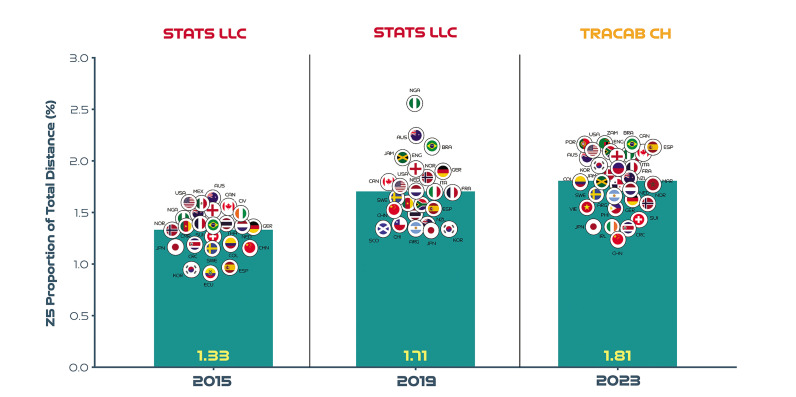
Sprinting team distance (Zone 5 [Z5], ≥23.0 km · h^-1^) as a proportion of total distance across the last three editions of the FIFA Women’s World Cup.

**FIG. 5B:**
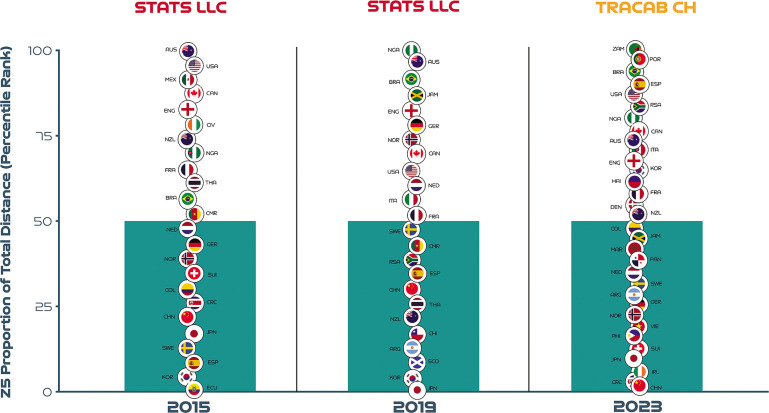
Sprinting team distance (Zone 5 [Z5], ≥23.0 km · h^-1^) as a percentile rank across the last three editions of the FIFA Women’s World Cup.

## DISCUSSION

The aims of the present study were to: (1) analyse the upper and lower match physical performance benchmarks and variability of teams at the FIFA Women’s World Cup Australia and New Zealand 2023, (2) examine the evolving team sprint ranking across three Women’s World Cups and (3) investigate noteworthy relationships between physical and tactical metrics at a team level.

The general trends highlighted the high match demands placed on contemporary female international teams. In that regard, tournament benchmarks for team physical performances were established for total distance covered (103 km), in addition to that covered at high-intensity (6.7 km) and sprinting (1.9 km). Some interesting team insights can be gleaned from the upper and lower ends of the competition’s physical performance continuum. For instance, Spain exhibited both volume and intensity characteristics during matches, while Vietnam were the exact opposite. Although physical capacities are closely related to the total and high-intensity distances covered by elite female players during matches, these relationships are complex [10]. Accordingly, fitness is not the only factor that determines a team’s physical output during match-play [12]. The submaximal nature of the sport usually requires players to exert themselves intensely during matches only when required [5]. A strong modulatory factor associated with the up or down regulation of match physical performances has been found to be the tactical scenarios players encounter [18, 19]. Thus, the style of play employed by each team combines with other factors, including the score, match importance and the standard of the opposition to influence physical outputs [12–13]. To support such an assertation, Vietnam’s low-intensity characteristics could be related to their limited ball possession and defensive style of play. This resulted in them frequently sitting in a compact defensive low- or mid-block for extended periods [20]. Similar match physical performance trends were reported at the FIFA Men’s World Cup 2022 for teams that employed a deep lying compact defence for long periods of the game [7]. In contrast, the high-intensity nature of Champions Spain could be linked to their dominance on the ball. Spain recorded the highest number of movements to receive and the second-greatest number of progression and final-third phase of play events at the tournament [20]. When Spain advanced, they regularly utilised the wide areas to cross or sought to penetrate through runs in behind the opposition’s defensive line, ranking first at the tournament for both [20]. Although Spain averaged the highest ball possession at the tournament, they still occasionally lost the ball and would intensely press, defensively transition and/or recover when required. Thus, the array of tactical activities above may require Spain’s players to utilise a high proportion of their physical capacities during games. This physicaltactical interdependency is understandable, as the aim of any team’s tactics is to ensure optimal organisation in order to make the most of their players’ capabilities [7]. Thus, these benchmarks are not only indicative of a team’s physical fitness but also reflect some of their stylistic tendencies.

To further contextualise the trends from the FIFA Women’s World Cup 2023, physical and tactical metrics were correlated to determine any noteworthy relationships. Interestingly, associations were found between the number of high-intensity runs that teams performed and various phase of play events that had urgency attached to their outcome (e.g., a risk of conceding or a chance of scoring). In particular, high-intensity efforts out-of-possession were related to the number of defensive recoveries and transitions a team produced. In support of this finding, teams have been found to cover the greatest proportion of their high-intensity distance out-of-possession performing recovery runs [19]. Due to the potential consequences of not tracking back, it is understandable that teams work intensely out-of-possession to rapidly recover into their defensive shape [7]. Interestingly, nations that were competing at the tournament for the first time (Philippines, Zambia and Panama) performed a high number of both intense efforts and defensive recoveries/transitions. This suggests that the least experienced teams found it challenging against more seasoned opponents and thus had to recover intensely more often. Similarly, high-intensity efforts in-possession were found to be related to the number of progression and final third events. This indicates that teams increase their intensity once they progress the ball forward via vertical passes or dribbles in an attempt to pose an attacking threat [7]. This ‘switching up’ of game tempo was particularly evident for highly offensive teams such as Brazil, Spain and the USA as opposed to deeper lying more defensive teams that spent more time out-of-possession such as Costa Rica, the Philippines and Vietnam. Research has revealed that the greatest proportion of a team’s high-intensity actions occur during fast transition-based activities [18, 19]. Thus, the correlations above could indicate that team’s produce a significant amount of their intense running to either recover defensively into a better shape or to get forward as part of an attack. However, the reader should view these associations with some caution as correlation does not necessarily equal causation.

The present study reveals for the first time the average match-tomatch variation of teams at the FIFA Women’s World Cup 2023. Research has found that the variability of physical metrics from a positional perspective was intensity-dependent in elite female players [11]. The current findings confirm this assertation but from a collective perspective as team match-to-match CV’s during the tournament were only 4% for the total distance but 11–19% for that covered at higher intensities. The match-to-match variation for the men’s teams at the FIFA World Cup 2022 was similar for the total distance covered (CV: 3%), but more stable at the higher intensities (CV: 9–14%) compared to their female counterparts [7]. Team trends for the stability of match physical performances at the FIFA Women’s World Cup 2023 were metric specific. Selected teams were more consistent match-to-match from a physical perspective for metrics such as total distance covered (Brazil), in addition to that covered at high-intensity (Italy) and sprinting (Panama). Notably, Costa Rica exhibited the most extreme match-to-match variation of the tournament for highintensity and sprinting distances. The opposition that Costa Rica played against at the upper and lower ends of their range provides muchneeded context. For instance, Costa Rica covered their greatest highintensity distances against Spain and Zambia, while their lowest was against Japan. Both Zambia and Spain were ranked first and second at the tournament for game intensity, while Japan was ranked in the bottom 6–8 teams, hence the extensive data spread. The interdependency of the factors involved in matches means that a team’s standard and intensity can have a profound impact on their opposition’s work rate [12, 13]. Given the multi-facetted and variable nature of football performance, the identification of factors that up or down regulate team physical outputs is incredibly challenging as the context changes considerably within and between games [7]. Thus, practitioners may need to focus their lens on game-by-game trends to gain a more holistic understanding of the impact of contextual factors on team physical outputs.

Benchmarking several tournaments or multiple seasons of a competition enables practitioners to map physical developments longitudinally [7, 14, 15]. Interestingly, team sprinting distance as a percentage of the total distance covered was found to be 29–36% higher in the FIFA Women’s World Cup 2019 and 2023 compared to the 2015 edition [17]. However, numerous changes across tournaments makes it difficult for researchers to track performance evolutions. Fortunately, the FIFA Women’s World Cup 2015 versus 2019 sprinting trends (29% increase in 4 years) are comparable due to the standardisation in tracking technology and game rules at both tournaments. Thus, this finding could be attributed to a physical performance ascent in the women’s game. Moreover, this finding aligns well with the evidence from men’s football that indicates an intensification of game demands over time [7, 14, 15, 23]. However, a comparative sprinting evaluation was not possible for the FIFA Women’s World Cup 2023 data set (36% increase in 8 years) due to a different tracking technology been employed at that tournament. Additional complexities that impact between tournament comparisons include new rule directives that enabled teams to make five substitutes in the FIFA Women’s World Cup 2023, as opposed to three in the 2015 and 2019 tournaments [24]. On average 5.6 (max: 6.0) versus 7.4 substitutions (max: 10) were made per game at the FIFA Women’s World Cup 2019 and 2023, respectively [25]. Interestingly, Champions Spain utilised their full quota of substitutes in five of their seven games in the 2023 tournament, highlighting the exceptional depth of their squad. Thus, this type of squad management could also have contributed to the greater sprint demands in the FIFA Women’s World Cup 2023 versus previous editions, as substitutes cover more sprint distance on a per minute basis compared to those starting the game or those that were replaced [26]. More second half substitutions may also account for the negligible between halve deficits observed for team high-intensity and sprinting metrics in the FIFA Women’s World Cup 2023. The present study was also able to shed some light on which teams increased, decreased or maintained their sprinting percentile rank across several tournaments. Notably, Champions Spain and China PR demonstrated the most pronounced percentile rank increase and decrease for sprinting across the last three FIFA Women’s World Cups, respectively. Although complex, these basic percentile rankings may provide practitioners with longitudinal insights into which countries have physically evolved more relative to others. This general upward shift in game intensity indicates modern international women’s teams are now expected to cover substantial distances at higher intensities. Thus, greater importance should be placed on training modalities that optimally prepare teams for the rigours of the modern women’s game [27, 28].

It is imperative that the myriad of limitations inherent in the present study are acknowledged. The physical data presented are one dimensional in nature as acceleration and change of direction metrics are not included. Moreover, the present study could not determine the between tournament evolution in physical performances due to technological differences and as a result only basic percentile rankings for each tournament could be analysed. Each optical tracking system has been found to be valid regarding the physical profiling of players [29, 30], and as such they should be able to physically rank teams appropriately in each tournament. Despite this, the reader should still view these trends with some caution. Thus, football organisations should attempt to standardise optical tracking systems longitudinally to ensure physical developments can be sufficiently monitored in future. The direct integration of physical and tactical metrics was not possible for this study. As a correlational approach was adopted, it is important for the reader to be cognisant that correlation does not equal causation when examining these trends. Finally, the trends observed in the present study would have benefitted from the inclusion of effective playing time and future research studies in this area are warranted.

## CONCLUSIONS

This study quantified the physical characteristics of teams competing at the FIFA Women’s World Cup 2023. The findings demonstrate the upper and lower benchmarks of contemporary international tournament football and the degree of variation exhibited by teams across multiple matches. Practitioners should be aware that the physical demands of teams are shaped by a myriad of factors and this makes interpretations particularly challenging. Although some interesting longitudinal benchmarking trends are presented, it is imperative that the reader views only the within tournament rankings (Figure 5B) as opposed to absolute sprint distances between tournament (Figure 5A) due to different systems. The general benchmarking trends revealed the high match demands placed on contemporary female international players. Thus, practitioners should prioritise high-intensity training modalities that also include technical, tactical and coordination components to fully prepare female players for the rigours of modern international women’s match-play [31, 32]. Specifically focusing on potent training modalities such as speed endurance training as research has continued to support not only its effectiveness but also its underlying adaptive mechanisms [33]. Finally, to gain a deeper understanding of women’s match demands, it is imperative that the reader links the team trends presented here with the positional trends observed at this tournament [34].
